# Polymeric Propranolol Nanoparticles for Intraocular Delivery: Formulation, Characterization, and Vitreous Pharmacokinetics

**DOI:** 10.18502/jovr.v19i1.15436

**Published:** 2024-03-14

**Authors:** Farkhondeh Chaharband, Reyhaneh Varshochian, Rassoul Dinarvand, Hamideh Sabbaghi, Mozhgan Rezaei Kanavi, Narsis Daftarian, Ramin Nourinia

**Affiliations:** ^1^Department of Pharmaceutics, Faculty of Pharmacy, Tehran University of Medical Sciences, Tehran, Iran; ^2^Department of Pharmaceutics, School of Pharmacy, Shahid Beheshti University of Medical Sciences, Tehran, Iran; ^3^Nanotechnology Research Center, Faculty of Pharmacy, Tehran University of Medical Sciences, Tehran, Iran; ^4^Department of Pharmaceutical Nanotechnology, Faculty of Pharmacy, Tehran University of Medical Sciences, Tehran, Iran; ^5^Ophthalmic Epidemiology Research Center, Research Institute for Ophthalmology and Vision Science, Shahid Beheshti University of Medical Sciences, Tehran, Iran; ^6^Ocular Tissue Engineering Research Center, Research Institute for Ophthalmology and Vision Science, Shahid Beheshti University of Medical Sciences, Tehran, Iran; ^7^Experimental Medicine, Department of Medicine, The University of British Columbia Faculty of Medicine, Vancouver, BC, Canada; ^8^Ophthalmic Research Center, Research Institute for Ophthalmology and Vision Science, Shahid Beheshti University of Medical Sciences, Tehran, Iran

**Keywords:** Ocular Neovascularization, Pharmacokinetic, PLGA Nanoparticles, Propranolol Vitreous

## Abstract

**Purpose:**

Recent studies have reported the promising effect of intravitreal propranolol on retinal neovascularization. However, rapid clearance and short half-life of the drug in the vitreous are the main drawbacks of this therapeutic approach. This study investigates the extension of the residence time of propranolol in the vitreous by polymeric nanoparticles (NPs) with the prospect of improving choroidal neovascularization treatment

**Methods:**

The poly (lactic-co-glycolic) acid (PLGA) NPs were fabricated by a modified double emulsion solvent evaporation method and the obtained NPs were characterized for their size, poly dispersity index (PDI), and surface image. The *in vitro* release, cell cytotoxicity, and uptake of NPs were also evaluated. To investigate the effect of the vitreous pharmacokinetic drug loaded NPs versus that of the free propranolol, they were intravitreally injected into the rabbits' eyes and the drug vitreous concentrations in defined intervals were analyzed by high performance liquid chromatography (HPLC).

**Results:**

The spherical NPs with about 230 nm size, and almost 10% drug loading were obtained. Based on the 3-(4, 5-Dimethylthiazol-2-Yl)-2, 5-Diphenyltetrazolium Bromide (MTT) outcomes, 30 µg/ml of propranolol was considered as the guide dosage in the intravitreal injection. Confocal microscopy images verified the presence of labeled NPs in the posterior segment after five days of receiving the injection. *In vivo* assay revealed that the vanishing rate of propranolol in rabbits treated with propranolol NPs was reduced at twice the rate as compared to that of the vanishing rate experienced with only the free drug.

**Conclusion:**

PLGA NPs can prolong the existence of propranolol in both vitreous and posterior ocular tissues, and thus, may provide an effective approach in treatment of posterior segment neovascularization.

##  INTRODUCTION

Choroidal neovascularization (CNV) is one of the main causes of visual loss particularly in the elderly population^[1,2]^ and is the leading cause of central vision loss in neovascular age-related macular degeneration (nAMD). The normal angiogenesis process is essential for wound healing and embryogenesis. However, pathological angiogenesis has “problematic adverse effects” which occur in many types of diseases such as cancer and retinopathies. Previous studies have indicated the role of the vascular endothelial growth factor (VEGF), the platelet-derived growth factor (PDGF), and the stromal derived factor (SDF-1) in pathological angiogenesis where amongst all three, VEGF is the most important factor.^[3,4,5,6,7,8]^ Therefore, deactivation of one or more of these factors, especially VEGF, can lead to therapeutic achievements.^[6,9,10,11]^

The angiogenesis inhibitory effects of propranolol, a nonselective β-adrenergic receptor blocking agent, were reported in previous studies.^[1,10]^ However, although its exact anti-angiogenesis mechanism is still unclear, reducing the proliferation, differentiation, and migration of endothelial cells, vasoconstriction, and inhibiting the overexpression of VEGF and basic fibroblast growth factor (bFGF) genes have been suggested. Systemic or intravitreal injection of propranolol was able to inhibit the CNV in animal models and may potentially suggest a new treatment for retinal and CNV in humans.^[1,2][3,4,5,6,7,8][9][10][11][12][13][14][13]^ Although vitreous pharmacokinetic of propranolol is still unknown, due to its small molecular size and hydrophilicity, the rapid clearance can be predictable and thus evaluating the extended-release formulations of the drug may be sensible. The current study was designed to enhance the intraocular local delivery of propranolol and decrease its probable systemic toxicity.

Due to the specific physiology and complexity of ocular barriers, such as blood–retina barrier (BRB), inner limiting membrane and vitreous, the management of posterior segment diseases is challenging. Current CNV treatments have considerable drawbacks including the possibility that repeated intravitreal injections may induce adverse effects such as hemorrhage, retinal detachment, and endophtalmitis and may threaten patients' quality of life. Novel drug delivery systems have been suggested to resolve these problems by enhancing the drug penetration into the various barriers and extending its residence time. According to previous studies, polymeric nanoparticles (NPs) may reduce the need for multiple intravitreal injections and may prevent some serious adverse effects.^[14,15,16,17]^ Poly (lactic-co-glycolic) acid (PLGA) is one of the most prevalent biodegradable and biocompatible polymers which has been approved by FDA for injectable formulations and has shown promising potentials in drug delivery.^[15,16,18]^ Furthermore, PLGA NPs can effectively overcome various ocular barriers and make ocular drug delivery more efficient.^[19]^

In this study propranolol-loaded PLGA NPs were prepared, characterized, and evaluated in terms of *in vitro* release. Moreover, the pharmacokinetics of free propranolol versus propranolol-loaded NPs in rabbit vitreous were investigated to reach a controlled release delivery system.

##  METHODS

### Preparation of Propranolol-loaded NPs

In our proposed method, propranolol was encapsulated in NPs via a modified double-emulsion solvent evaporation process. 0.5 ml of propranolol (gifted by TolidDaru Company, Tehran. Iran) solution in water (1 mg/ml) was emulsified in 1.25 ml of organic phase solution dichloromethane (DCM; Merck, Darmstadt, Germany) containing PLGA (RG502H, glycolide:lactide ratio of 50:50, Boehringer Ingelheim, Ingelheim Germany), by sonicating for 2 min using a probe sonicator (Misonix, microtip 419, 15 W). In the next step of sonication, 2.5 ml of poly vinylalcohol 1% aqueous solution (PVA; PVA sterile eye drops, Sina Daru, Tehran, Iran) was added to the primary emulsion and the mixture was sonicated for a further 3 min (20 W). All sonication steps were done in an ice bath. The final emulsion (W/O/W) was stirred over night at 500 rpm at room temperature to allow the evaporation of the excess organic phase and the hardening of the NPs. The final nano suspension was subsequently centrifuged (14000 rpm, 4ºC) to collect the prepared NPs. Finally, the prepared NPs were lyophilized.

### Characterization of Propranolol-loaded NPs

The mean diameter and the PDI of NPs were determined by dynamic light scattering (DLS) (Zetasizer Nano ZS, Malvern, UK). The size and morphology of the prepared NPs were observed by emission scanning electron microscopy (FESEM) (LEO1455VP).

To calculate the entrapment efficiency of the NPs, an indirect technique was employed.^[20]^ Accordingly, during the NP preparation the obtained nano-suspension was centrifuged (14000 rpm, 4 C, 15 min) and the clear supernatant was collected and analyzed using the HPLC method. The percentage of entrapment efficiency (EE%) was determined by the following equation: 


EE%=[(totalamountofpropranolol−amountofpropranololinsupernatant)÷totalamountofpropranolol]×100.

To determine the drug loading percentage (DL%) for NPs, the lyophilized NPs were weighed and then the equation below was used. 


DL%=[(totalamountofpropranolol−amountofpropranololinsupernatant)÷nanoparticlemass]×100.

### In vitro Drug Release 

To examine the release profile of propranolol from formulated NPs, they were suspended in phosphate buffer saline (P pH = 7.4) and placed in the shaker incubator at 37ºC and 250 rpm, then in specified intervals, certain amounts of the samples were collected and fresh PBS was added in order to maintain the sink condition. All samples were centrifuged (14000 rpm, 4ºC, 15 min) in order to determine the released profile of the drug by applying the HPLC method.

**Table 1:** Pharmacokinetic parameters of free propranolol and propranolol NPs.


	**Free Propranolol**	**NP**
AUC (μg.min.ml ${\;}^{-1}$ )	5029.55	11932.36
AUMC (μg.min^2^.ml ${\;}^{-1}$ )	1164078.22	3498043.48
MRT (min)	231.45	293.16
K (min ${\;}^{-1}$ )	0.0042	0.0033
CL (ml.min ${\;}^{-1}$ )	0.0060	0.0025
	
	
AUC, area under the curve; AUMC, area under the first moment curve; MRT, minimum residence time; K, The average elimination rate constant; CL, clearance; NP, nanoparticles		

**Figure 1:**
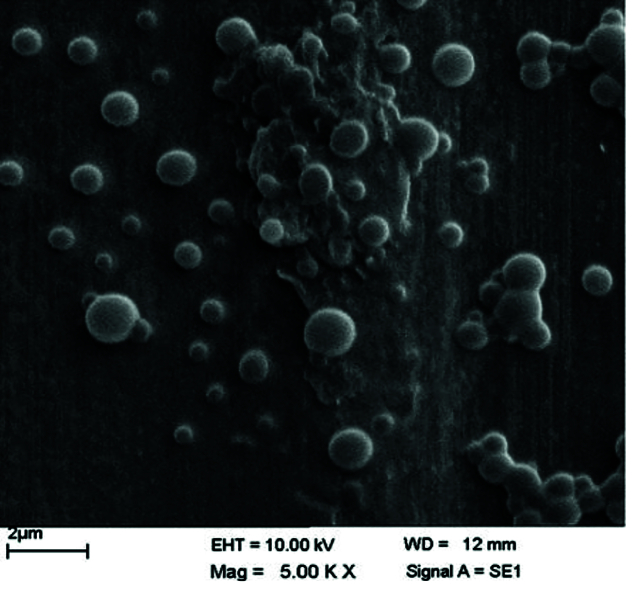
FESEM image of propranolol NPs. As demonstrated, the NPs successfully designed with spherical shape and acceptable size and distributions.

**Figure 2:**
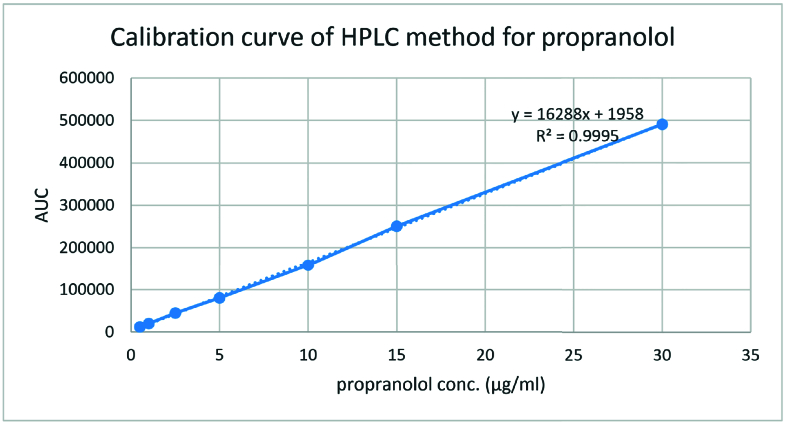
HPLC calibration curve for propranolol analysis in aqueous medium. The obtained calibration curve was plotted to calculate the free propranolol concentration in aqueous humor.

**Figure 3:**
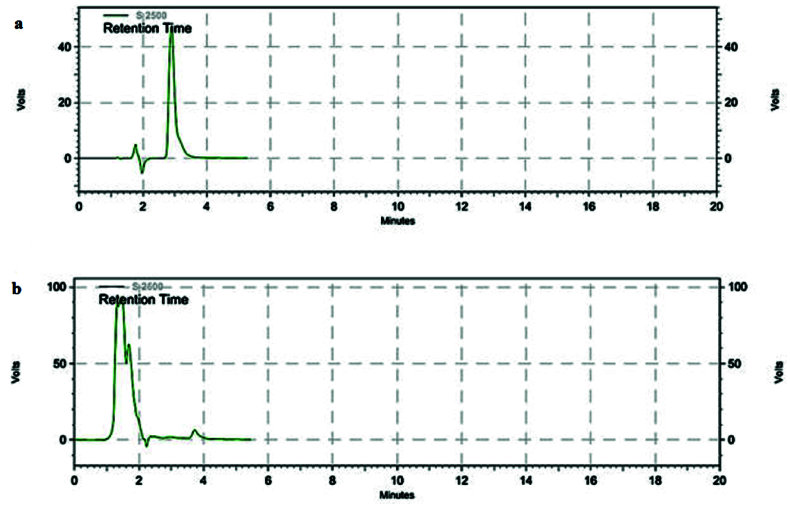
Propranolol HPLC chromatogram. (a) HPLC Chromatogram of propranolol in aqueous medium. As demonstrated, the “a” retention time of propranolol in aqueous medium is about 3 min for (b) HPLC Chromatogram of propranolol in vitreous. The retention time for propranolol in vitreous medium is about 4 min.

**Figure 4:**
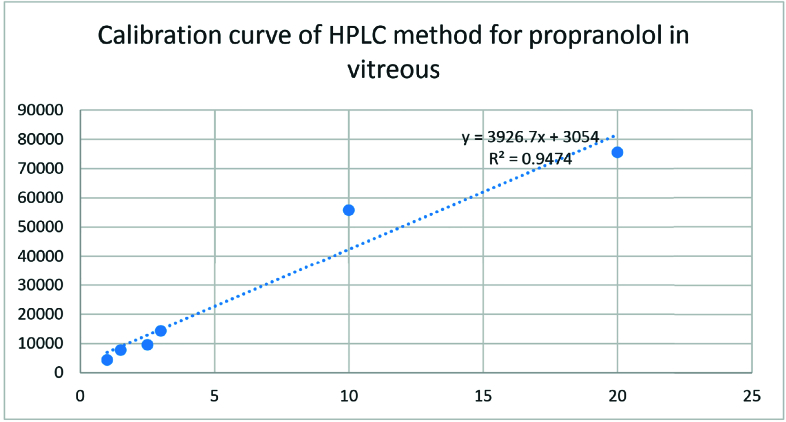
HPLC calibration curve for propranolol in vitreous. The obtained calibration curve was plotted to calculate the free propranolol concentration in vitreous.

**Figure 5:**
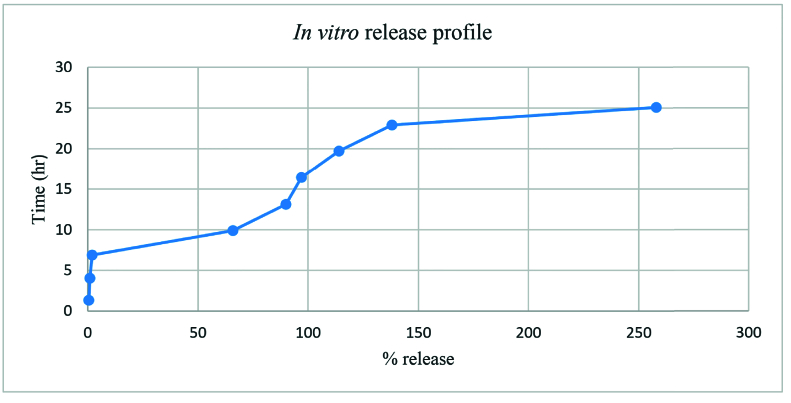
Release profile of propranolol from NPs in PBS, pH 7.4. The represented graph shows that the release of propranolol from PLGA NPs starts from primary minutes, reaches to the maximum content after 4 hr, and then reaches to the plateau state at the concentration of 16 µg/ml.

**Figure 6:**
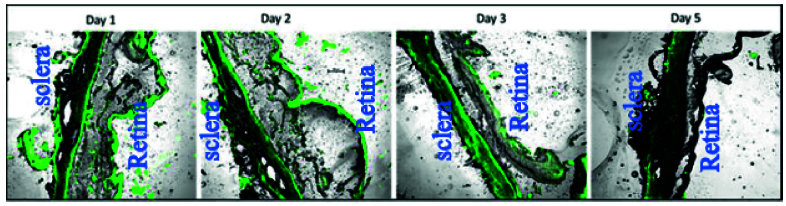
MTT assay results. Effect of various concentrations of free propranolol, free PLGA NPs, and propranolol NPs on cell viability after 24, 48, and 72 hr. Viability was determined as the percentage of living cells in treated cultures to control. Note that the maximum safe dose of propranolol is about 30 µg and an appropriate final concentration of PLGA is about 52 µdilution. The different samples concentration were calculated on the base of propranolol.

**Figure 7:**
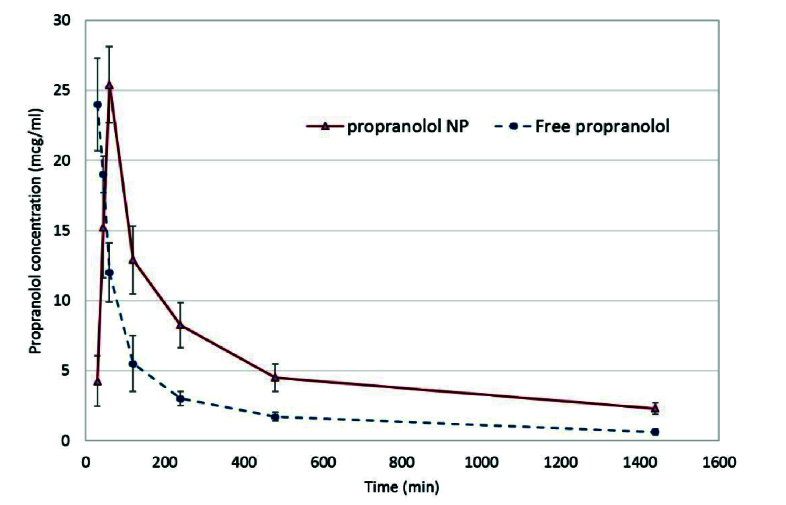
Confocal microscopy images of FITC labeled NPs in posterior eye segment. The distribution and persistence of NPs in posterior ocular tissues after 24, 48, 72, and 120 hr, representing the continuous release of propranolol during the test span.

**Figure 8:**
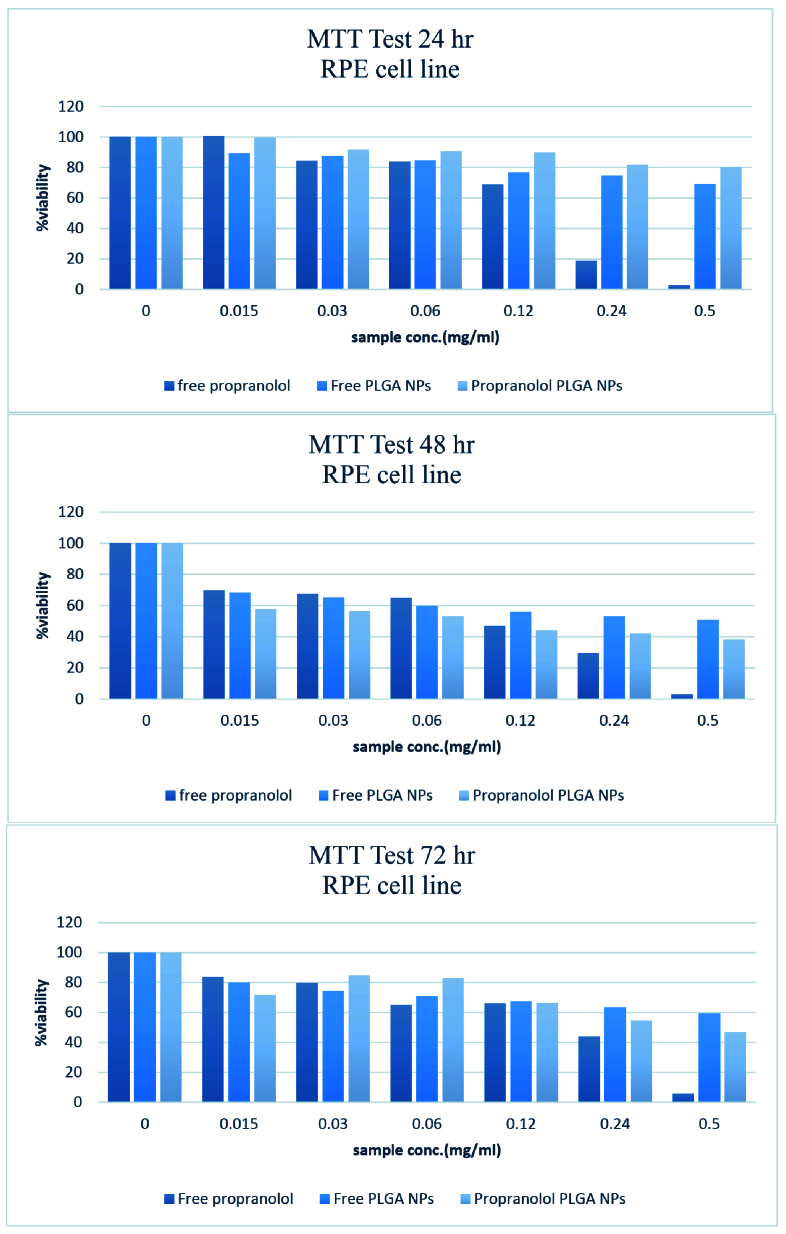
The amount of free propranolol (a) and released propranolol from nanoparticles (b) at different time intervals in rabbit vitreous after intravitreal injection. Note the twice vanishing rate of free propranolol as compared to the propranolol nanoparticles.

### HPLC Analysis of Propranolol

The HPLC method was developed to determine the propranolol concentration *in vitro* and in the rabbit vitreous. The stationary phase used was a 25 × 0.46 cm C18 column with a particle size of 5 μm mounted on a Kneuer HPLC system (Berlin, Germany) consisting of a Well chrome K1001 pump, a Rheodyne injector armed with a 20 µL sample loop, and a K2600 UV detector. Propranolol was detected at 280 nm. The mixture of acetonitrile/KH ${\;}_{2}$ PO ${\;}_{4}$ (0.02 M, pH: 4.3) (40:60, v/v) (Merck, Darmstadt, Germany) was utilized as the mobile phase with a flow rate of 1.5 ml/min.

Calibration curve was schemed for propranolol solutions with concentrations of 0.5, 1, 2.5, 5, 10, 25 and 30 µg/ml in water. Accuracy was calculated by analyzing triplicate samples from six calibration standards with the aforementioned concentrations and was stated as relative error, RE%, calculated with the following equation:

RE% = ([absolute difference between calculated and spiked concentration] / calculated concentration) *100.

Precision values were determined by analyzing three replicates of the calibration standards and was expressed as the relative standard deviation (RSD %).^[20]^

### Extraction Procedure

To determine the concentration of released propranolol from NPs in the vitreous, the collected samples from rabbits' eyes were first extracted. In our proposed extraction method, methanol (Merck, Darmstadt, Germany) was considered as an extraction solvent. In this procedure, 500 µl methanol was added to 300 µl spiked vitreous sample and then stirred for 3 min. Afterward, the mixture was centrifuged (7500 rpm, 20 min, 4ºC) and the clear supernatant was injected into the HPLC system. The calibration curve in the vitreous was plotted for propranolol solutions with different concentrations of 1, 1.5, 2.5, 3, 10, and 20 µg/ml.

### In vitro Cytotoxicity Assay

In order to determine the *in vitro* cytotoxicity of the formulated NPs, the 3-(4, 5-Dimethylthiazol-2-Yl)-2, 5-Diphenyltetrazolium Bromide (MTT) (Sigma-Aldrich Co. St Louis, MO, USA) assay was performed. Cell viability was assayed using equivalent dosages of free propranolol, drug-free PLGA NPs, and propranolol-loaded NPs on the cultivated human retinal pigment epithelium (RPE) cells (Ocular Tissue Engineering Research Center, Shahid Beheshti University of Medical Sciences, Tehran, Iran.), where untreated cells were considered as controls. The RPE cells were maintained in a Dulbecco's Modified Eagle Medium DMEM enrichment with 20% fetal bovine serum (FBS) and 2 mM L-glutamine at 37ºC in a 5% CO ${\;}_{2}$ incubator. Accordingly, 10,000 cells/well were located in 96-well plates and treated with the mentioned samples. After 2 hr, the medium was substituted with a fresh culture medium. The test was completed after 24, 48, and 72 hr and the colorimetric determination of cell viability was performed and relative growth prevention was calculated at 540 and 630 nm. All experiments were performed in triplicate.

### Tissue Uptake Evaluation

To evaluate the posterior tissue uptake of NPs, FITC (Sigma-Aldrich Co. St Louis, MO, and USA) was loaded into NPs instead of propranolol. New Zealand White rabbits were divided into three groups and the FITC NPs were injected intravitreally. Each group was enucleated at specified times after injection (24, 48, 72, and 120 hr). Then cryosections were prepared from eyeballs using a microtome (6µm) and the corresponding posterior tissues were analyzed by a confocal microscope (Nikon, Eclipse TIE, and Japan).

### Pharmacokinetic Assay

The study was performed on 14 New Zealand adult rabbits. Rabbits were divided into two groups, one for NPs and the other for free propranolol. Animals were anesthetized by administering a mixture of ketamine (10 mg/kg body weight) and xylazine (5 mg/kg body weight). Then 30 µg of free propranolol and NPs containing equal amounts of drug and 52 µg PLGA were intravitreally injected in 14 healthy rabbits. Subsequently, at specific time intervals, the vitreous samples were collected in order to determine the vitreous pharmacokinetic parameters of propranolol. In all steps, propranolol assay was done by HPLC. The test was performed in triplicate.

### Statistical Analysis

Statistical analysis, including *t*-test and one-way ANOVA were performed using the Excel 2016 and Sigma Plot. Results were considered statistically significant at a *P*-value < 0.05.

##  Results

### Characterization and quantification of propranolol loaded NPs

According to the results of the DLS, the mean diameter of the designed NPs was about 230 nm with a PDI of 0.12. The scanning electron microscopy (SEM) image of the NPs showed that the NPs were spherical and in accordance with DLS results in size and distribution [Figure 1]. The loading and entrapment efficiency of NPs were calculated by the stated formula in the previous section and the results were 10 ± 4% and 70 ± 12%, respectively.

### In vitro Release of NPs

The HPLC analysis method was validated by the propranolol solutions with various concentrations and each concentration was repeated in triplicate on three different days. The obtained calibration curve was plotted to calculate the free propranolol concentration [Figure 2]. The retention time of propranolol was about 3 min for aqueous samples and nearly 4 min for vitreous samples [Figure 3a & 3b]. The chromatogram of the blank sample proved that the HPLC method was selective for propranolol analysis. All the validation processes for analysis of propranolol in the aqueous solution were repeated for the vitreous samples [Figure 4].

Among various organic solvents such as methanol, acetonitrile, and ethyl acetate, methanol showed the highest recovery (about 70%), and proved that the procedure was successful in performing drug extraction from the vitreous.

The drug release profile from the NPs in PBS showed that the release of propranolol started from the early minutes, reached the maximum content after 4 hr, and then moved to the plateau state at the concentration of 16 µg/ml [Figure 5].

### MTT Assay

The MTT assay was utilized to determine the safe concentration of free propranolol, drug-free PLGA NPs, and propranolol-loaded PLGA NPs. As demonstrated in Figure 6, the maximum safe dose of propranolol was 30 ± 0.02 µg and an appropriate final concentration for both free PLGA NPs and propranolol-loaded NPs was about 52 µg based on the PLGA concentration.

### Tissue Uptake Evaluation

Results of posterior segment uptake of FITC-loaded NPs after 24, 48, 72, and 120 hr is shown in Figure 7. The obtained images indicated that NPs were distributed and persisted in the posterior segment tissues after 24, 48, 72, and 120 hr which might present the continuous release of propranolol during the test span.

### Propranolol Pharmacokinetics in the Rabbit Vitreous

Figure 8 shows the vitreous concentration of propranolol at varied time intervals following intravitreal injections of free propranolol versus drug-loaded NPs in rabbits. Non-compartmental methods were employed to determine the vitreous pharmacokinetic parameters of propranolol regardless of a particular compartmental model. The basic calculations were based on the area under the drug vitreous concentration versus time curve (AUC) and the area under the moment curve (AUMC). The AUC was calculated using the trapezoidal rule. The AUMC is the area under the concentration and time versus time curve. AUMC was also determined by the trapezoidal rule. The final segment of the AUC curve was calculated as Cv(last point)/k, where k was the last exponential. The last segment for the AUMC curve was obtained from Cv(last point)*t (last point)/k+ Cv(last point)/k^2^.

The mean residence time (MRT) was obtained from AUMC/AUC, which was the average time that the propranolol stayed in vitreous. It was related to the average elimination rate constant (K) as 1/MRT. The drug clearance from the vitreous was also calculated as CL = Dose/AUC.

In Table 1, the calculated pharmacokinetic parameters are summarized.

As illustrated, MRT of propranolol in rabbits treated with propranolol-loaded NPs increased by 27% compared to that in the free drug rabbits (*P* = 0.035). Therefore, the elimination rate constant and the clearance rate decreased to 21% and 58%, respectively (*P* = 0.009 and 0.003, respectively).

##  DISCUSSION

In this study, the vitreal pharmacokinetics of propranolol and PLGA NPs containing propranolol following intravitreal injection in rabbits was investigated, and to the best of our knowledge, was reported for the first time.

It has been shown that propranolol, a nonselective β-blocker agent, inhibits ocular neovascularization via reducing proliferation, migration, and differentiation of endothelial cells, and inhibiting the VEGF and VEGFR2 signaling pathways. In addition, it has been reported that propranolol and other β-blockers may suppress angiogenesis by reducing the insulin growth factor-1 (IGF-1) mRNA, hypoxia-inducible factor-1 (HIF-1), and VEGF levels.^[21]^ Tahiri et al reported a significant reduction in VEGF levels and CNV growth following the daily intraperitoneal propranolol injection (6 mg/kg/d) in mice for 10 days.^[21]^ To achieve improved efficacy and less side effects, intravitreal administration was chosen instead of a systemic route. In one of the previous studies performed by Nourinia et al, safety of intravitreal injections of propranolol for CNV treatment was reported. Accordingly, 0.3 µg and 30 µg per eye were deemed safe in mice and rabbits, respectively.^[1]^ Moreover, the promising therapeutic effect of intravitreal propranolol on retinal capillary hemangioma (RCH) in a patient with Von Hippel–Lindau was reported by Karimi et al. Propranolol 50 mg/0.05 mL was injected intravitreally twice six weeks apart; no significant adverse effect was observed during the study period.^[22]^ However, the intravitreal pharmacokinetics and the drug residence time in vitreous were not previously studied. Due to the small molecular size and hydrophilicity and the short MRT, the rapid clearance of propranolol from the vitreous was predictable. The steep descending slope and rapid clearance observed in the current *in vivo* results [Figure 8] following intravitreal injection of free propranolol confirmed this assumption. To address this issue according to the previous experiences,^[23]^ the PLGA nano particulate system was considered to prolong the drug vitreal residence.

The PLGA NPs were prepared by a double emulsion solvent evaporation method and 230 nm NPs with spherical shape with no remarkable aggregation obtained. Multiple studies have indicated that the main advantage of the double emulsion method is being one of the best techniques for incorporation of a hydrophilic agent-like propranolol into a hydrophobic polymer such as PLGA.^[23]^ It should be noted that the PVA solution volume and concentration, the second step of sonication, in addition to the rate of PVA addition and the power of sonication were the most important factors in NPs formulation to reach an appropriate size and PDI. The loading and entrapment efficiency of formulated NPs were calculated and the results were in an acceptable range being comparable with similar PLGA NPs.^[23]^

The release test was employed to determine the free propranolol in the PBS at different time intervals. As demonstrated in Figure 5, the amount of drug (propranolol), which was released from the polymeric NPs, was raised after 4.16 hr and then it reached a plateau state. It seems that the small size of propranolol molecules affected the release profile and propranolol was released rapidly through the NPs. Furthermore, the erosion and degradation rate of PLGA NPs also played a role in the drug release procedure.

The MTT assay was employed to determine the possible toxic effect of formulated NPs on cultivated human RPE cells. As expected and according to the PLGA specifications as a biocompatible and biodegradable polymer, the obtained results after 24, 48, and 72 hr proved that the designed carrier and the propranolol NPs were safe and showed no significant toxicity on cultured RPE cells in the purposed concentrations.

Ocular tissue uptake of the fluorescent NPs was examined after the intravitreal injection of the formulated NPs in rabbits. Confocal microscopic examinations disclosed the presence of NPs in the posterior segment of the eye which means that the drug release and effect can be continued for nearly one week.

*In vivo* experiments results confirm that the half-life of the drug in NPs was more that of the free propranolol. As demonstrated in Figure 8, the descending slope of propranolol concentration released from NPs was slower than that of the free drug.

Altogether, the designed NPs were able to improve the *in vivo* behavior and vitreous pharmacokinetics of propranolol, and the obtained results showed a statistically significant increase in MRT value, this elevation may not clinically be helpful. The rapid clearance of NPs can be explained by NP diffusion in vitreous and more retinal uptake. Moreover, vitreous macrophages can also be involved in NP eliminations. Indeed, *in vivo* studies on CNV cases can reveal the exact potential of the designed NPs. Incorporation of NPs into the *in-situ* gel-forming polymers or implant may result in more benefits, which needs further investigations.

In summary,the vitreous pharmacokinetics of free propranolol versus propranolol-loaded PLGA NPs following the intravitreal injection was studied in rabbits. The NPs were formulated by a modified double emulsion solvent evaporation method. The *in vivo* results indicated that the retinal uptakes of NPs resulted in an elongated persistence of propranolol in both vitreous and posterior ocular tissues and thus, may provide an effective approach in posterior segment neovascularization treatment. Determination of drug concentration in the various eye segments can be considered as the future plan.
